# Helios expression and Foxp3 TSDR methylation of IFNy+ and IFNy- Treg from kidney transplant recipients with good long-term graft function

**DOI:** 10.1371/journal.pone.0173773

**Published:** 2017-03-15

**Authors:** Karina Trojan, Christian Unterrainer, Rolf Weimer, Nuray Bulut, Christian Morath, Mostafa Aly, Li Zhu, Gerhard Opelz, Volker Daniel

**Affiliations:** 1 Transplantation-Immunology, Institute of Immunology, University Hospital Heidelberg, Heidelberg, Germany; 2 Department of Internal Medicine, University of Giessen, Giessen, Germany; 3 Department of Nephrology, University of Heidelberg, Heidelberg, Germany; 4 Nephrology unit, Internal Medicine Department, Assiut University, Assiut, Egypt; 5 Department of Hematology, Tongji Hospital, Huazhong University of Science and Technology, Wuhan, China; INSERM, FRANCE

## Abstract

**Background:**

There is circumstantial evidence that IFNy+ Treg might have clinical relevance in transplantation. IFNy+ Treg express IFNy receptors and are induced by IFNy. In the present study we investigated in kidney transplant recipients with good long-term stable graft function the absolute cell counts of IFNy+ Treg subsets and whether their expression of Foxp3 is stable or transient.

**Method:**

Helios expression determined by eight-color-fluorescence flow cytometry and methylation status of the Foxp3 Treg specific demethylation region (TSDR) served as indicators for stability of Foxp3 expression. Methylation status was investigated in enriched IFNy+ and IFNy- Treg preparations originating from peripheral blood using high resolution melt analysis. A total of 136 transplant recipients and 52 healthy controls were studied.

**Results:**

Proportions of IFNy+ Treg were similar in patients and healthy controls (0.05% and 0.04% of all CD4+ lymphocytes; p = n.s.). Patients also had similar absolute counts of IFNy producing Helios+ and Helios- Treg (p = n.s.). Most of the IFNy+ and IFNy- Treg in transplant recipients had a methylated Foxp3 TSDR, however, there was a sizeable proportion of IFNy+ and IFNy- Treg with demethylated Foxp3 TSDR. Male and female patients showed more frequently methylated IFNy+ and IFNy- Treg than male and female controls (all p<0.05).

**Conclusions:**

Kidney transplant recipients with good long-term stable graft function have similar levels of IFNy+ Treg as healthy controls. IFNy+ and IFNy- Treg subsets in patients consist of cells with stable and cells with transient Foxp3 expression; however, patients showed more frequently methylated IFNy+ and IFNy- Treg than controls. The data show increased levels of Treg subsets with stable as well as transient Foxp3 expression in patients with stable allograft acceptance compared to healthy controls.

## Background

Long-term treatment of organ transplant recipients with immunosuppressive drugs can cause severe side-effects, such as an increased risk of life-threatening infection or cancer [1]. Immunosuppressive treatment strategies are therefore being developed to minimize these side-effects. In-vivo induction or the administration of in-vitro expanded regulatory T-cells (Treg) might be an effective option [2, 3]. A goal of Treg therapy is the induction or expansion of Treg subsets with maximal stability of phenotype and immunosuppressive function. As shown by other authors, there is a risk that Treg differentiate to Th1 and Th17 lymphocytes during inflammation [4]. During the process of reprogramming they may lose Foxp3 expression and immunosuppressive activity, and they may even gain potency to attack the graft [4–6]. It has been reported that thymus-derived Treg (tTreg) possess greater immunosuppressive stability than peripherally formed Treg (pTreg) or in-vitro induced Treg (iTreg) [7].

Recently, we reported that a particular subset of the Treg pool, IFNy+ Treg, might have clinical relevance [8]. IFNy+ Treg express IFNy receptors and are induced by IFNy [9, 10]. They might represent the first line of peripherally induced pTreg or activated thymically-derived tTreg. They patrol through the body, search for IFNy and initial immune responses, and inhibit effector cells specifically and unspecifically by secretion of TGFß and IL10, consumption of IL2 and IFNy, as well as CD152-mediated cell-cell interaction. Moreover, this subset is able to kill effector cells by perforin/granzyme B or CD178-induced apoptosis, is susceptible to manipulation by immunosuppressive drugs in-vitro, and appears to be rather stable with respect to reprogramming [5, 8–14]. Recently, Sela et al. reported on dendritic cells that are able to induce a subpopulation of IL-12Rß2 expressing Treg that specifically consume IL12 to control Th1 responses [15]. These cells produce, in addition, IFNy [15]. IFNy+ Treg were shown to be stronger immunosuppressive in-vitro than IFNy- Treg [9, 11].

In the present study we investigated the absolute cell counts of IFNy+ Treg subsets in the peripheral blood of renal transplant recipients with stable good long-term graft outcome, whether they are mainly thymus-derived tTreg or peripherally induced pTreg, and whether their expression of Foxp3 is stable or transient. Demethylation of the Treg-specific demethylated region (TSDR) of Foxp3 [16, 17] and up-regulation of the transcription factor Helios [18–20] modulate and stabilize Foxp3 expression. Helios is expressed on the majority of thymus derived tTreg [21], however, part of thymus derived tTreg lack Helios expression [22]. Peripherally induced pTreg do not express Helios [21] but are able to up-regulate Helios in combination with Foxp3 during cell activation [23, 24]. Thymus derived tTreg were shown to have stable Foxp3 expression, in contrast to peripherally induced pTreg which present Foxp3 only transiently with the risk of reprograming from Treg to Th1 effector cells [4, 5]. Stability of Foxp3 expression that is linked to the immunosuppressive function of Treg would be desirable for their use in diagnosis and therapy of transplant recipients [5]. Identification of the dominating Treg subset in patients with good long-term stable graft function was thought to offer valuable clues as to which Treg subset should be induced in-vivo or expanded in-vitro for therapeutic use and posttransplant monitoring.

## Methods

### Patients and healthy control individuals

All patients and controls gave informed consent for the tests performed within this study and the study was approved by the Heidelberg ethical committee (S-225/2014). The study was conducted in adherence to the Declaration of Helsinki. All participants provided their written informed consent to participate in this study. This consent procedure was approved by the local ethics committee. Laboratory staff and healthy blood donors served as healthy controls (n = 52). Blood from renal transplant recipients (n = 136) was obtained during regular visits in the outpatient clinics of the university hospitals Heidelberg and Giessen. Second and third blood samples were obtained in 3 months intervals from 59 and 11 patients, respectively. Demographic data of the patients are summarized in Table 1. Blood levels of cyclosporine, tacrolimus and mycophenolate were available in 21, 104 and 44 patients, respectively.

**Table 1:** Demographic data of patients and healthy control individuals.

Category	Patients (n, %)	Healthy Controls (n, %)
**Sex:**		
**male**	91 (67%)	26 (50%)
**female**	45 (33%)	26 (50%)
**Age, mean, years**	54±14	41±12
**<25**	1 (1%)	3 (5.8%)
**25–64**	98 (72%)	49 (94%)
**>64**	37 (27%)	-
**Graft No.:**		
**first**	115 (85%)	-
**second**	16 (12%)	-
**third**	5 (4%)	-
**Donor:**		
**living**	54 (40%)	-
**deceased**	82 (60%)	-
**Serum creatinine, mg/dl:**		
**<2**	97 (71%)	-
**≥2**	39 (29%)	-
**GFR, ml/min:**		
**<60**	101 (74%)	-
**≥60**	35 (26%)	-
**Immunosuppressive protocol**		
**steroids**	82 (60%)	-
**cyclosporine**	20 (15%)	-
**tacrolimus**	104 (76%)	-
**mycophenolate mofetil**	91 (67%)	-
**azathioprine**	15 (11%)	-

### Determination of PBL subsets

PBL subsets were determined as described previously and the exact gate setting inclusively FACS plots were described in a recent publication [13]. For analysis of determinants on the cell surface, PBL were incubated with fluorochrome-labelled monoclonal antibodies against CD45, CD3, CD4, CD8, CD16, CD56, CD19, CD25 and CD127 (all from BD Biosciences). Intracellular determinants were stained with fluorochrome-labelled monoclonal antibodies against Foxp3, IFNγ (clone B27) and Helios (ebioscience, Frankfurt, Germany). Briefly, PBL were incubated with combinations of monoclonal antibodies for 30 min as described and eight-color fluorescence was analyzed using a FACSCanto II triple-laser flow cytometer (BD Biosciences) [13]. When, in addition, intracellular proteins were studied, cell membranes were permeabilized using BD Perm/Wash buffer (BD Biosciences). At least 100,000 events were analyzed in the initial FSC/SSC dot plot. IFNγ monoclonal antibody used for cell separation (BD clone 4S.B3) and IFNγ monoclonal antibody used for cell staining (BD clone B27) were not competitive (data not shown).

### Enrichment of CD4+CD25+CD127-IFNγ+ Treg

IFNy+ Treg were selected on the basis of surface-bound IFNy. Using this procedure, CD4+CD25+CD127-IFNγ+ Treg were enriched to >80% in the IFNy+ Treg fraction whereas IFNy- Treg preparations contained <20% IFNy+ Treg [9, 11]. CD4^+^ and CD25+ PBL were subsequently separated by positive selection using Strep-Tactin magnetic Microbeads (IBA, Göttingen, Germany) according to the instructions of the manufacturer. Briefly, 4 μl Fab-Strep were mixed with 1μl Buffer IS. Then, 15 μl from the homogeneously resuspended Strep-Tactin Magnetic Microbeads were added to the Fab-Strep solution and incubated overnight at 4°C. The pre-incubated Fab-Strep Microbead preparation was added to the cells and mixed gently for 20 min at 4°C. Thereafter, 5 ml of buffer IS was added to the cell/bead preparation. Magnetically labelled cells were separated by placing the tube for 3 min onto the StrepMan magnet. The negative cell fraction was removed with the supernatant whereas the positive cell fraction was flushed off the tube surface by resuspension in 5 ml buffer IS. Magnetic selection was repeated twice. Labelled cells were resuspended in 10 ml of a 1 mM D-biotin working solution and incubated for 10 min at room temperature. The tube was placed on the StrepMan magnet for 3 min and supernatant containing the target cell fraction was pipetted off and transferred to another tube. The last step was repeated twice. Cells were centrifuged by 400 g for 10 min and resuspended in buffer. Using this procedure, CD4+ and CD25+ PBL were enriched. CD127- PBL were separated by negative selection and IFNγ+ PBL were separated by subsequent positive selection, as described previously [9, 11]. Cells were incubated with CD127 monoclonal antibody (BD Biosciences, Heidelberg, Germany) and CD4^+^CD25^+^CD127^-^ were separated from CD4^+^CD25^+^CD127^+^ cells using streptavidin-coupled beads (Dynabeads Biotin Binder, Invitrogen, Dynal Oslo, Norway) according to the instructions of the manufacturer. The procedure was repeated using IFNγ monoclonal antibody (BD Biosciences, Heidelberg, Germany, IFNγ clone 4S.B3).

### DNA Isolation

Because of limited blood sample material for Treg subset isolation, determination of Foxp3 TSDR methylation status was not possible with both primers in IFNy+ and IFNy- Treg preparations of every patient blood sample. Genomic DNA of separated CD4+CD25+CD127-IFNγ+ and CD4+CD25+CD127-IFNγ- T cells was extracted using the QIAamp cultured cells Mini Kit (Qiagen, Hilden, Germany) according to the protocol of the manufacturer. DNA was stored in Eppendorf tubes (Eppendorf, Hamburg, Germany) at -20°C.

### Bisulfite conversion of genomic DNA

Bisulfite converts unmethylated cytosines to uracil, whereas methylated cytosines remain unreactive. After conversion of unmethylated cytosines to uracil by bisulfite treatment and subsequent PCR-mediated conversion of uracils to thymine, methylated and unmethylated alleles differ in thermal stability because of their different CpG contents. Bisulfite conversion of genomic DNA was performed using the EZ DNA Methylation Kit (Zymo research, Freiburg, Germany) according to the manufacturer’s instructions, as described previously [13]. CT Conversion Reagent powder was dissolved in 750 μl water and 210 μl M-Dilution buffer. 5 μl of M-Dilution buffer was added to the isolated DNA samples. Total volume was adjusted to 50 μl with water and mixed. CT Conversion Reagent and DNA samples were incubated separately in the dark at 37°C for 15 min. After incubation, 100 μl of CT Conversion Reagent was added to each DNA sample. Samples were mixed and incubated in the dark at 50°C for 12–16 hours. After incubation DNA samples were placed on ice for at least 10 min and then loaded into a Zymo-Spin IC Column containing 400 μl M-Binding buffer, mixed, and centrifuged at 10 000 g for 30 sec. Additional 100 μl of M-Wash buffer were added to the column and centrifuged at 10 000 g for 30 sec. 200 μl of M-Desulphonation buffer were added to the column, incubated at room temperature for 20 minutes and centrifuged at 10 000 g for 30 sec. Another 200 μl of M-Wash buffer were added to the column and centrifuged at 10 000 g for 30 sec and the procedure was repeated. Thereafter, the Zymo-Spin IC Column was placed into a clean 1.5 ml microcentrifuge tube (Eppendorf), 15 μl of M-Elution buffer were added to the column matrix, and samples were centrifuged at 10 000 g for 30 sec. DNA was eluted in Eppendorf tubes and stored at -20°C for later use.

### Foxp3 TSDR DNA methylation analysis

HRM analysis after bisulfite treatment identifies methylation variations in the Foxp3 gene. Bisulfite modified DNA was subjected to PCR according to the protocol for HRM analysis provided by the primer manufacturer (EpigenDx, Hopkinton, MA, USA), as described previously [13]. ADS3576 primers were used for the amplification of the promoter and 5’UTR region, and ADS783 primers for the amplification of the intron 1 TSDR of the Foxp3 gene. Primer P1 (ADS783; 181 bp; -2376 to -2263 from ATG) covered 11 and primer P2 (ADS3576; 231 bp; -6216 to -6278 from ATG) 5 CpGs sites, as described in the manual of the manufacturer. PCR was carried out in a 21 μl total volume containing: 2.1 μl PCR buffer, 0.27 μl MgCl_2_, 0.42 μl dNTPs, 1.05 μl SYBR Green dye (EpigenDx), 1.26 μl each of primers ADS783 and ADS3576 (Human Foxp3 Methylation Panel, EpigenDx), 0.12 μl Hot Start Taq polymerase (Qiagen), and 1.5 μl of bisulfite-treated genomic DNA (concentration 5 ng/μl). The amplification protocol with ADS783 primers consisted of incubation at 95°C for 15 min followed by 45 cycles at 95°C for 30 sec, 62°C for 30 sec and 72°C for 30 sec with a final extension step at 72°C for 5 min. The amplification protocol with ADS3576 primer consisted of an incubation at 95°C for 15 min followed by 45 cycles at 95°C for 30 sec, 59°C for 30 sec and 72°C for 30 sec with a final extension step at 72°C for 5 min. Melting was performed from 60°C to 90°C at a melt rate of 1%. Each sample was analyzed in triplicate. DNA methylation analysis and diagram generation was performed using real-time PCR software (Applied Biosystems 7500, Foster City, CA, USA).

### Standard curve

7 commercially available standards with different proportions of methylated Foxp3 template DNA were used to estimate methylation status of the samples (0%, 5%, 10%, 25%, 50%, 75%, 100%) (Human Methylation Controls—Mix, EpigenDx). Templates were pretreated with bisulfite. 75% was the second last standard of the 7 standards provided by the manufacturer and this standard appears to represent an appropriate cut-off for the discrimination of patients with strong Foxp3 TSDR methylation from those with intermediate or low Foxp3 TSDR methylation.

### Statistics

Statistical analysis was performed using PASW Statistics program version 21 (IBM, Chicago, Illinois, USA) and Wilcoxon rank sum and Mann-Whitney U test. P-values <0.01 were considered significant (Bonferroni correction).

## Results

### Patients

**Fig 1:**
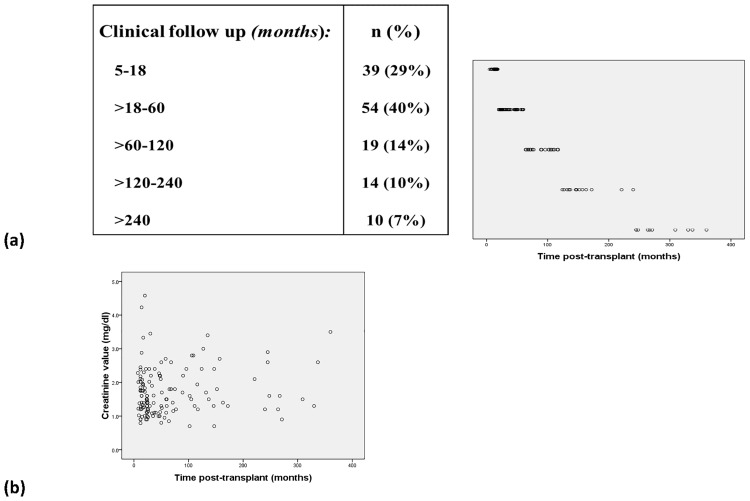
Graft function of long-term stable kidney transplant recipients. Fig 1a shows the number of transplant recipients and Fig 1b their serum creatinine levels with respect to time posttransplant.

IFNy+ Treg were studied in 136 transplant recipients 5 months to 360 months posttransplant (mean±SD: 1946±2201 days) (Fig 1a). Only 6 of them had a serum creatinine >3 mg/dl whereas the majority (71%) showed creatinine levels < 2 mg/dl (Fig 1b). All patients had good and stable long-term graft function, received low immunosuppressive maintenance drug doses and had no current acute rejections or infections. Fifty-two healthy individuals served as controls. Further demographic data are presented in Table 1.

### Lymphocyte subsets and total Treg

**Fig 2:**
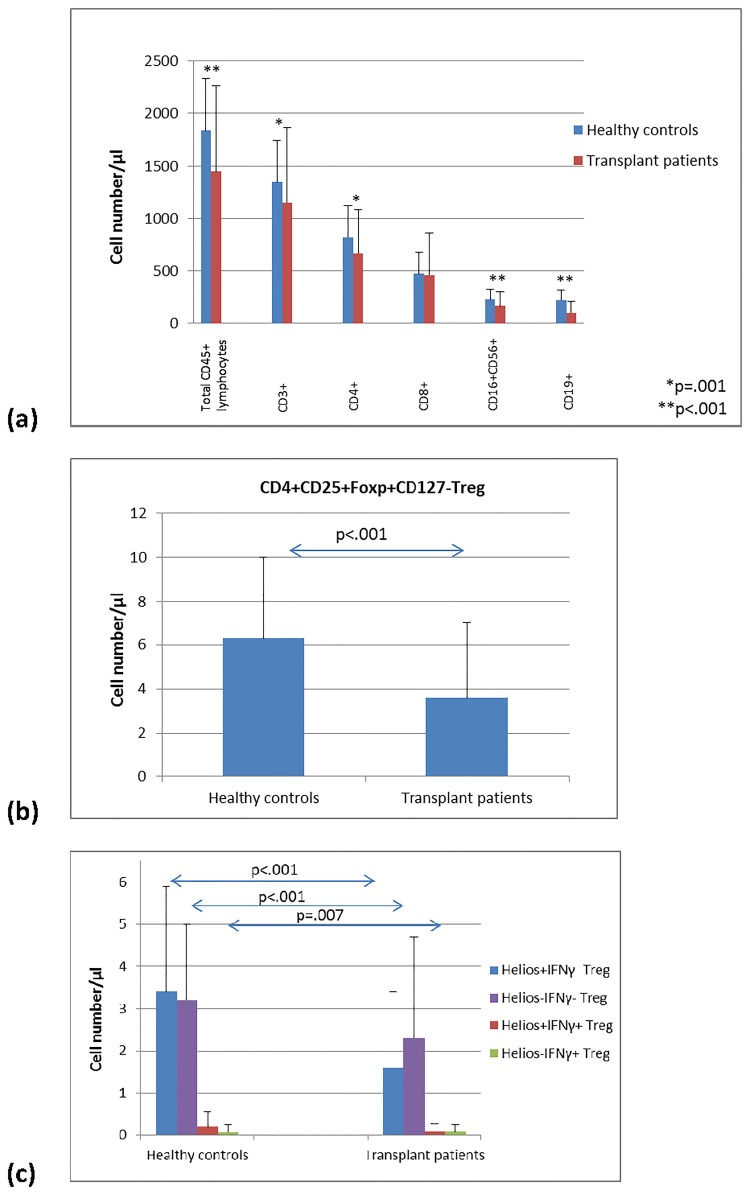
Lymphocyte and Treg subsets in long-term stable kidney transplant recipients. Long-term stable kidney transplant recipients (n = 136) have lower absolute counts of (a) CD45+, CD3+, CD4, CD16+56+ and CD19+ lymphocytes, (b) CD4+CD25+Foxp3+CD127- Treg and (c) Helios+IFNy-, Helios-IFNy- and Helios+IFNy+ Treg subsets than healthy controls (n = 52) (all p<0.01, Mann-Whitney U test). Data are given as mean±SD.

Patients had lower absolute numbers of CD45+ total T lymphocytes, CD3+ and CD4+ T cells, CD19+ B lymphocytes and CD16+CD56+ NK cells (all p≤0.01; Mann-Whitney U test) than healthy controls whereas CD8+ T cells were only borderline significantly lower in patients (p = 0.045) (Fig 2a). In addition, patients showed lower CD4+CD25+Foxp3+CD127- Treg cell counts than healthy controls (p<0.001) (Fig 2b). Most of the Treg were Helios+ or Helios- Treg without IFNy production and both subsets were lower in transplant recipients than healthy controls (p<0.001) (Fig 2c). IFNy+ Treg represent only a small proportion of circulating Treg and we found significantly lower absolute numbers of Helios+IFNy+ (p = 0.007) and similar Helios-IFNy+ Treg counts (p = 0.586) in patients compared to healthy individuals (Fig 2c). Absolute numbers of Helios+IFNy+ and Helios-IFNy+ Treg were similar in patients (p = 0.174) and were also similar in healthy controls (p = 0.197). Interestingly, the proportion of IFNy+ Treg (Helios+ plus Helios-) was similar in patients and controls with 0.05% and 0.04% IFNy+ Treg of total Treg, respectively. The data indicate that IFNy+ Treg are Helios+ or Helios- and may originate from thymus or periphery with stable or transient Foxp3 expression, that absolute cell counts of IFNy+Helios+ and IFNy+Helios- Treg are similar in the blood of patients, and that the proportion of IFNy+ Treg is similar in patients and healthy individuals.

When 59 transplant recipients were investigated a second time 120±47 days (mean±SD) after the first investigation, and 11 patients a third time 106±19 days after the second investigation, there was no statistically significant difference among lymphocyte and Treg subsets (1^st^ vs 2^nd^ and 2^nd^ vs 3^rd^ investigation; Wilcoxon signed rank test; p>0.01; data not shown) suggesting stability of lymphocyte and Treg absolute cell counts late posttransplant.

### IFNy+ Treg, graft function and immunosuppressive protocol

IFNy+Helios+ as well as IFNy+Helios- Treg were not associated with GFR or serum creatinine in long-term stable transplant recipients (p>0.01; Spearman rank correlation test). Daily doses of steroids (n = 82 patients), cyclosporine (n = 20), tacrolimus (n = 104), and mycophenolate mofetil (n = 92) as well as blood levels of cyclosporine (n = 21 patients), tacrolimus (n = 104) and mycophenolate mofetil (n = 44) were not associated with T-, B- and Treg subset absolute numbers in the blood (p>0.01; Spearman rank correlation test).

### Methylation of Foxp3 TSDR in enriched IFNy+ and IFNy- CD4+CD25+CD127- Treg

**Fig 3:**
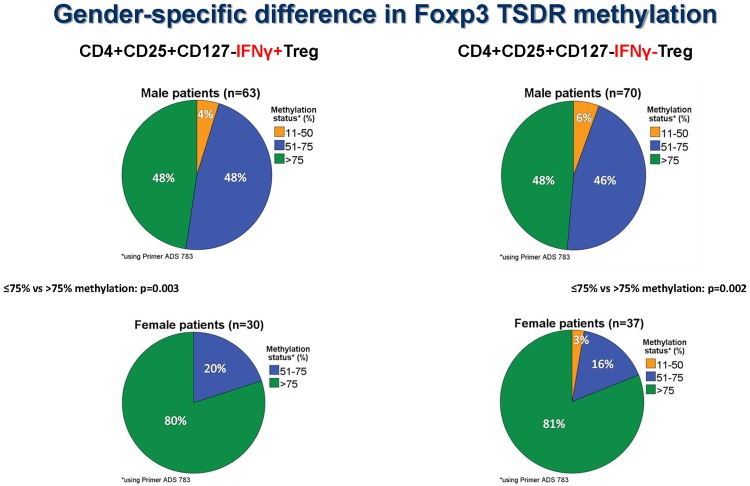
Gender specific differences in Foxp3 TSDR methylation. Female transplant recipients have more frequently methylated (>75% Foxp3 TSDR methylation) enriched IFNy+ and IFNy- CD4+CD25+CD127- Treg than male transplant recipients (p<0.003 and p<0.002, Mann-Whitney U test).

**Fig 4:**
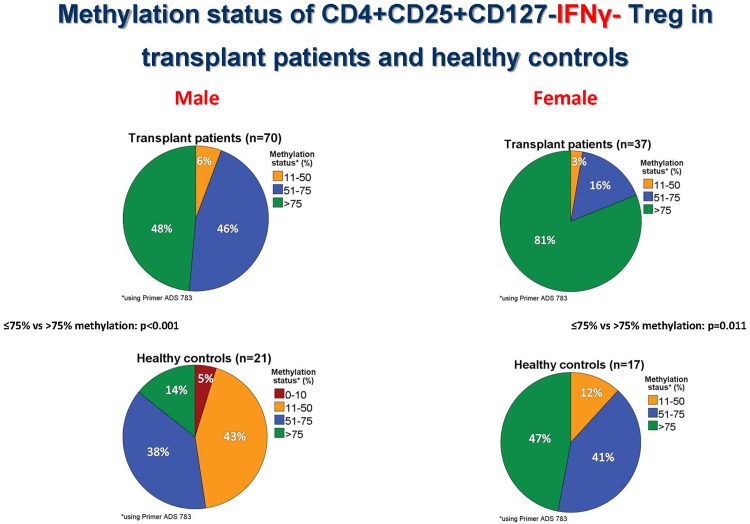
Methylation status of CD4+CD25+CD127-IFNγ+ Treg in transplant patients and healthy controls. Approximately, half of male kidney transplant recipients with good long-term stable graft function show mainly methylated (>75% Foxp3 TSDR methylation) IFNy+ Treg in the blood whereas the other half of male patients possess in addition a sizeable proportion of demethylated (11–75% Foxp3 TSDR methylation) IFNy+ Treg suggesting that they possess IFNy+ Treg with transient as well as stable Foxp3 expression.

**Fig 5:**
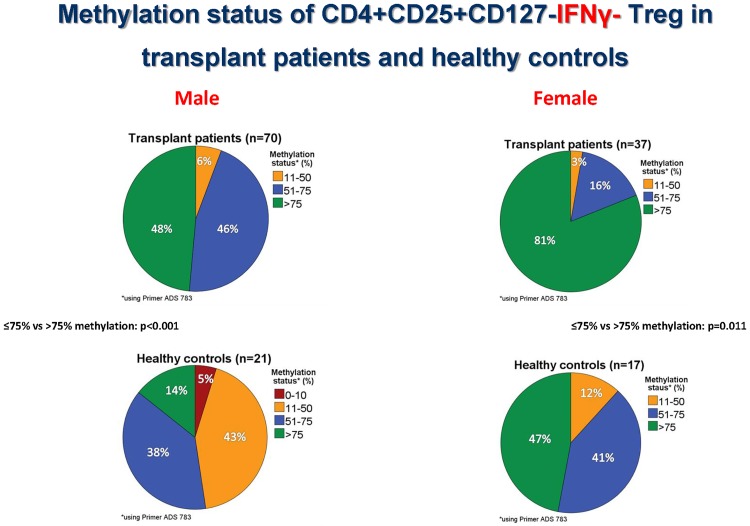
Methylation status of CD4+CD25+CD127-IFNγ- Treg in transplant patients and healthy controls. Approximately, half of male kidney transplant recipients with good long-term stable graft function show mainly methylated (>75% Foxp3 TSDR methylation) IFNy- Treg in the blood whereas the other half of male patients possess in addition a sizeable proportion of demethylated (11–75% Foxp3 TSDR methylation) IFNy- Treg suggesting that they possess IFNy- Treg with transient as well as stable Foxp3 expression. Interestingly, male and female transplant patients have more frequently methylated IFNy- Treg (>75% Foxp3 TSDR methylation) than healthy controls (p<0.001 and p = 0.011, respectively, Mann-Whitney U test).

Figs 3–5 show the Foxp3 TSDR methylation status obtained with primer P1 (ADS783), S1 Table exhibits, in addition, the results of primer P2 (ADS3576).

Because Foxp3 is located on the x-chromosome, Foxp3 TSDR methylation was studied separately in males and females using enriched IFNy+ and IFNy- CD4+CD25+CD127- Treg preparations. Female patients showed a higher proportion of IFNy+ (p = 0.003) as well as IFNy- Treg with >75% TSDR methylation than male patients (p = 0.002), confirming the stronger methylation of Treg in females than males (Fig 3). Interestingly, 30 male (48%) and 24 female (80%) patients possessed IFNy+ Treg with >75% methylation, suggesting that half of the male and four-fifths of the female patients had predominantly methylated IFNy+ Treg in the circulation. Another 30 male (48%) and 6 female patients (20%) showed IFNy+ Treg preparations with intermediate methylation (51–75% Foxp3 TSDR methylation), indicating a sizable proportion of demethylated Treg in the circulation of these patients. There were 3 male (4%) and no female patients with 11–50% methylated IFNy+ Treg which suggests predominantly demethylated IFNy+ Treg (Fig 4). Male (p<0.001) and female patients (p = 0.011) had more frequently methylated IFNy- Treg (>75% Foxp3 TSDR methylation) than male and female controls (Fig 5) whereas this association was not observed for IFNy+ Treg (>75% vs ≤75% Foxp3 TSDR methylation: p = 0.631 and p = 0.786, respectively) (S1 Table). Using a second primer for the determination of the Foxp3 TSDR methylation status (primer P2, ADS3576) male and female patients showed more frequently methylated IFNy+ (>75% vs ≤75% Foxp3 TSDR methylation: p = 0.02 and p = 0.005, respectively) and IFNy- Treg (>75% vs ≤75% Foxp3 TSDR methylation: p = 0.085 and p = 0.006, respectively) than male and female controls (S1 Table). The data suggest that most of the IFNy+ Treg express Foxp3 transiently, but that a sizeable proportion of IFNy+ Treg shows stable Foxp3 expression in renal transplant recipients with stable good long-term graft function and that male and female patients show more frequently methylated IFNy+ and IFNy- Treg than male and female controls.

In 23 patients the Foxp3 TSDR methylation status was studied a second time, 104±40 days (mean±SD) after the first investigation. There was no statistically significant difference between the values obtained in the first and second investigation (p>0.01) (S1 Table), suggesting stability and reproducibility of Foxp3 methylation in clinically stable transplant recipients.

### Methylation of Foxp3 and graft function

**Fig 6:**
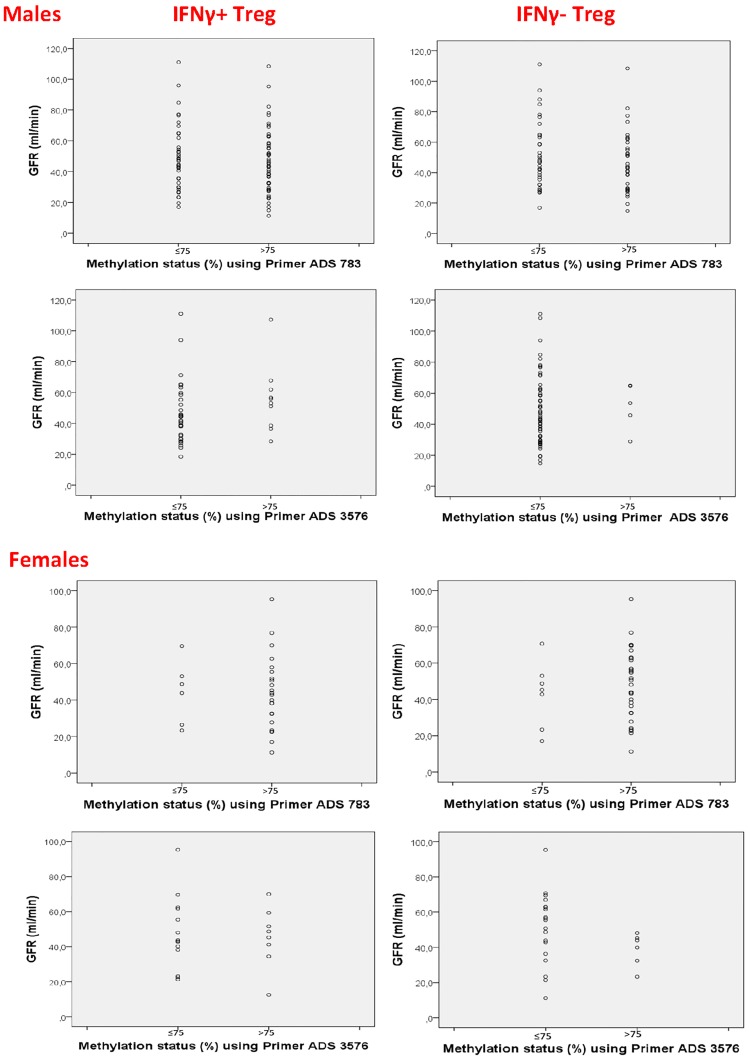
Methylation status of enriched IFNy+ and IFNy- CD4+CD25+CD127- Treg and graft function. There was no difference in GFR of patients with predominantly methylated (>75% Foxp3 TSDR methylation) Treg and patients with a sizable proportion of demethylated (≤75% Foxp3 TSDR methylation) Treg (p>0.01; Mann-Whitney U test). Enriched IFNy+ and IFNy- Treg of male and female patients were tested separately using primer ADS783 (primer P1) and primer ADS3576 (primer P2).

Patients with ≤75% Foxp3 TSDR methylation showed similar GFR and serum creatinine than patients with >75% methylation (p>0.05). Fig 6 shows the results for GFR and S2 Table the results for serum creatinine. The data suggest that GFR was not associated with the methylation level of Foxp3 TSDR in patients with good long-term graft function.

### Previous rejection/infection and Foxp3 TSDR methylation of IFNγ+ and IFNγ- Treg

Neither previous rejection nor previous viral or bacterial infection were associated with the Foxp3 TSDR methylation status of enriched IFNy+ or IFNy- Treg (p>0.05; Fisher´s exact test) (S3 and S4 Tables).

## Discussion

In the present study we determined the absolute cell counts of IFNy+ Treg subsets in renal transplant recipients with stable good long-term graft outcome and we looked whether these Treg have stable or transient Foxp3 expression as determined by Helios expression and Foxp3 TSDR methylation status.

Our data show that lymphocyte subsets including total Treg are decreased in long-term transplant recipients compared to healthy controls. Helios+ and Helios- Treg that lack IFNy production dominate the peripheral circulating Treg pool. However, there is a sizeable fraction of Helios+ and Helios- Treg in the blood that co-express IFNy and the proportion of IFNy+ Treg (Helios+ and Helios-) was similar in patients and healthy controls. In long-term stable transplant recipients with good graft function there is apparently no extraordinary IFNy secretion that would stimulate the formation of IFNy+ Treg. Consequently, these patients have normal proportions of IFNy+ Treg in the circulation. One might speculate that IFNy+ Treg are induced when they are needed. A recent publication reports that, contrary to expectation, overall proportions of donor-reactive Treg are similar before and after lung transplantation and increase during acute A2 cellular rejection of lung allografts, suggesting a mechanism how A2 rejection might be self-limiting [25]. The authors noted that the observed increases over baseline donor-reactive Treg were insufficient to prevent acute lung allograft rejection [25]. These data, together with ours, imply that Treg are induced during immune responses and have the power to modulate these immune responses. However, they do not entirely prevent immune responses against alloantigens. This would be a logical reason why transplant recipients with long-term stable graft function need low dose maintenance immunosuppressive drugs for prevention of acute rejection. Treg, and particularly IFNy+ Treg when induced during immune responses, might help to lessen the need for potent immunosuppression with drugs. However, during graft quiescence they remain low and are unable to prevent rejection efficiently in the absence of low dose immunosuppressive treatment.

Half of IFNy+ Treg in the circulation of transplant recipients are Helios+ and the other half Helios-, suggesting that IFNy+ Treg might originate from thymus as well as periphery [21, 22]. They might represent activated tTreg as well as peripherally induced pTreg. Expression of the transcription factor Helios controls Treg function [18–20, 26]. Expression of Helios by Tregs ensures stable expression of a suppressive and anergic phenotype in the face of intense inflammatory responses, whereas Helios deficient Treg display diminished lineage stability, reduced Foxp3 expression, and production of proinflammatory cytokines [27]. Nakagawa et al. reported recently that instability of Helios-deficient Treg is associated with conversion to a T-effector phenotype and enhanced antitumor immunity [27]. Thus, Helios expression of IFNy+ Treg might indicate stable and effective suppressive function. Analysis of the Foxp3 TSDR methylation status of enriched IFNy+ Treg supports the finding obtained by Helios phenotyping. Most patients had >50% methylated Treg irrespective of the primer used for the determination of methylation (S1 Table). However, approximately half of male and four-fifth of female patients showed >75% methylation of IFNy+ Treg, suggesting that in transplant recipients the pool of IFNy+ Treg consists of methylated as well as demethylated Treg. We studied methylation of cytosine residues in 2 different areas of the TSDR region in intron 1 of the Foxp3 gene. Primer P1 covered 11 CpG sites whereas primer P2 included 5 CpG di-nucleotides, as indicated by the manufacturer and reported in a recent publication [28]. The difference in CpG sites might account for the partly different results of our methylation analyses obtained with the 2 primers. However, as shown in S1 Table, the results obtained with both primers indicate that the majority of male and female patients and healthy controls have IFNy+ and IFNy- Treg with >50% Foxp3 TSDR methylation. The majority of male patients possess Treg with a methylation level of 51–75%, indicating that a sizable proportion of IFNy+ and IFNy- Treg is demethylated. Further analysis showed that higher GFR and lower serum creatinine levels were observed in patients with predominantly methylated Treg (>75% Foxp3 TSDR methylation) as well as in patients possessing a sizable proportion of demethylated Treg (≤75% Foxp3 TSDR methylation), suggesting that GFR was not associated with the methylation level of Foxp3 TSDR in patients with good long-term graft function.

It has been reported that methylated Treg induced peripherally in vivo (pTreg) or in-vitro (iTreg) suppress alloreactive effector cells, have transient Foxp3 expression, only weak immunosuppressive ability and have the potency to reprogram further to Th1 and Th17 lymphocytes during inflammation [4–6]. When PMA/Ionomycin as a stimulus was removed from cell cultures performed with lymphocytes from healthy individuals, the risk of reprogramming was low because induction of Treg continued for 96h after removal of the stimulus [13]. Based on these in-vitro experiments and the observation of consistently increased methylated Treg in transplant recipients with good long-term stable kidney function, we believe that the risk of methylated Treg to reprogram to harmful Th1 and Th17 lymphocytes is low in these patients. We believe that increased methylated Treg in combination with demethylated Treg contribute to good long-term graft function. However, further studies comparing patients late posttransplant with stable and unstable graft function are needed to differentiate these patient populations. Moreover, additional investigations of Treg subsets in lymph nodes and in graft biopsies have to clarify whether our observations in the peripheral blood of renal transplant recipients with stable long-term graft function are also detectable in lymph nodes and in the graft itself.

As observed by Hall et al. in experiments with tTreg obtained from rats and mice, long-term cell culture of tTreg with allogeneic stimulator cells in the presence of IL12 and/or IFNy generates an IFNy+ Th1-like Treg subpopulation that has a 100- to 1000-fold increased capacity to suppress alloresponses as compared to the original tTreg subset [29]. Such a Treg subset which suppresses alloresponses strongly, specifically and long-lasting would be ideal for clinical treatment of transplant recipients with Treg, and it would also be suitable for posttransplant Treg monitoring.

## Conclusions

Kidney transplant recipients with good long-term stable graft function do not show an up-regulation of IFNy+ Treg compared to healthy controls, and we speculate that during graft quiescence there is no increased IFNy stimulus for up-regulation of these cells. IFNy+ as well as IFNy- Treg of these patients exhibit stable as well as transient Foxp3 expression as determined by both Foxp3 TSDR methylation status and Helios expression. We speculate that Treg subsets with stable as well as transient Foxp3 expression contribute to stable allograft acceptance. The finding that methylated IFNy+ and IFNy- Treg are higher in patients than controls supports this speculation. Further studies comparing patients with stable good long-term graft function late posttransplant with those who experienced rapid deterioration of graft function late posttransplant because of immunologically induced rejection are needed to provide conclusive evidence for this hypothesis. Moreover, additional investigations of Treg subsets in lymph nodes and in graft biopsies have to clarify whether our observations in the peripheral blood of renal transplant recipients with stable long-term graft function can be confirmed in lymph nodes and in the graft itself.

## Supporting information

S1 Table Methylation status of Foxp3 TSDR in patients and healthy control individuals. (DOCX)

S2 Table Association of GFR and serum creatinine with Foxp3 TSDR methylation status of enriched IFNγ+ and IFNγ- Treg using primers P1 and P2. (DOCX)

S3 Table Association of viral and bacterial infection with Foxp3 TSDR methylation status of enriched IFNγ+ and IFNγ- Treg using primers P1 and P2. (DOCX)

S4 Table Association of rejection with Foxp3 TSDR methylation status of enriched IFNγ+ and IFNγ- Treg using primers P1 and P2. (DOCX)

## Abbreviations

AZA: Azathioprine
CD40L: CD40 ligand
CSA: Cyclosporine A
CTLA-4: cytotoxic T-lymphocyte-associated protein 4
Fab: fragment antigen binding
HRM: high resolution melt
IFNy: Interferon-gamma
IL: interleukin
iTreg: in-vitro induced T regulator cell
MMF: Mycophenolate mofetil
PBL: peripheral blood lymphocytes
PCR: polymerase chain reaction
PD-1: programmed cell death 1
pTreg: peripheral-derived T regulator cell
Tbet: T-box expressed in T-cells
TGFß: Transforming growth factor-beta
Th1: T helper type 1
Treg: regulatory T-cell
TSDR: Treg-specific demethylated region
tTreg: thymical-derivel T regulator cell
