# Correction: Helios expression and Foxp3 TSDR methylation of IFNy+ and IFNy- Treg from kidney transplant recipients with good long-term graft function

**DOI:** 10.1371/journal.pone.0179069

**Published:** 2017-05-31

**Authors:** Karina Trojan, Christian Unterrainer, Rolf Weimer, Nuray Bulut, Christian Morath, Mostafa Aly, Li Zhu, Gerhard Opelz, Volker Daniel

Fig 4 is incorrect. The authors have provided a corrected version here.

**Fig 4:**
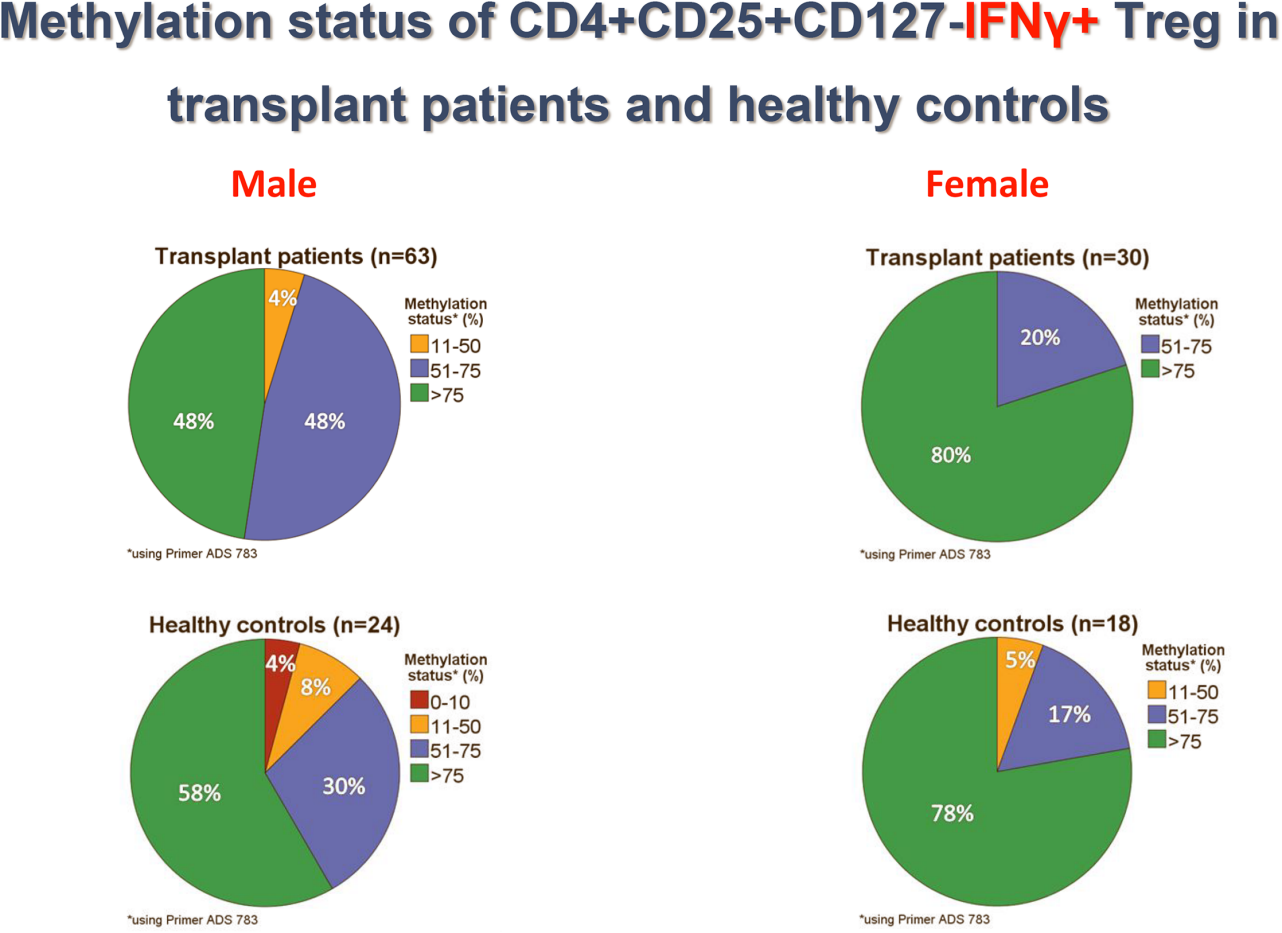
Methylation status of CD4+CD25+CD127-IFNγ+ Treg in transplant patients and healthy controls. Approximately, half of male kidney transplant recipients with good long-term stable graft function show mainly methylated (>75% Foxp3 TSDR methylation) IFNy+ Treg in the blood whereas the other half of male patients possess in addition a sizeable proportion of demethylated (11–75% Foxp3 TSDR methylation) IFNy+ Treg suggesting that they possess IFNy+ Treg with transient as well as stable Foxp3 expression.
