# Supplementary material for: Helios expression and Foxp3 TSDR methylation of IFNy+ and IFNy- Treg from kidney transplant recipients with good long-term graft function
Source: PLoS One. 2017 Mar 15;12(3):e0173773. doi: 10.1371/journal.pone.0173773 (PMC5351987; doi:10.1371/journal.pone.0173773)
Supplement: S1 Table — (DOCX) [file pone.0173773.s001.docx]

| **Foxp3 TSDR methy-lation** | **Healthy**  **individuals** | | **Patients**  **(first investigation)** | | **Patients**  **(second investigation)** | | **P*** | | | | | |
| --- | --- | --- | --- | --- | --- | --- | --- | --- | --- | --- | --- | --- |
|  | **Male**  (Group 1)  n= | **Female**  (Group 2)  n= | **Male**  (Group 3)  n= | **Female**  (Group 4)  n= | **Male**  (Group 5)  n= | **Female**  (Group 6)  n= | **Group**  **1 vs 2** | **Group**  **3 vs 4** | **Group**  **1 vs 3** | **Group**  **2 vs 4** | **Group**  **3 vs 5** | **Group**  **4 vs 6** |
| **P1IFNγ+** | | | | | | | | | | | | |
| **>75%** | 14 | 14 | 30 | 24 | 7 | 8 | 0.183 | 0.003 | 0.631 | 0.786 | 0.655 | 0.564 |
| **51-75%** | 7 | 3 | 30 | 6 | 7 | 1 |  |  |  |  |  |  |
| **11-50%** | 2 | 1 | 3 | 0 | 0 | 0 |  |  |  |  |  |  |
| **0-10%** | 1 | 0 | 0 | 0 | 0 | 0 |  |  |  |  |  |  |
| **P1IFNγ-** | | | | | | | | | | | | |
| **>75%** | 3 | 8 | 34 | 30 | 5 | 5 | 0.007 | 0.002 | <0.001 | 0.011 | 1.0 | 1.0 |
| **51-75%** | 8 | 7 | 32 | 6 | 4 | 1 |  |  |  |  |  |  |
| **11-50%** | 9 | 2 | 4 | 1 | 1 | 1 |  |  |  |  |  |  |
| **0-10%** | 1 | 0 | 0 | 0 | 0 | 0 |  |  |  |  |  |  |
| **P2IFNγ+** | | | | | | | | | | | | |
| **>75%** | 0 | 1 | 11 | 8 | 1 | 4 | 0.413 | 0.269 | 0.020 | 0.005 | 0.317 | 0.083 |
| **51-75%** | 2 | 10 | 30 | 14 | 5 | 0 |  |  |  |  |  |  |
| **11-50%** | 2 | 3 | 2 | 0 | 2 | 0 |  |  |  |  |  |  |
| **0-10%** | 0 | 1 | 0 | 0 | 0 | 0 |  |  |  |  |  |  |
| **P2IFNγ-** | | | | | | | | | | | | |
| **>75%** | 0 | 1 | 5 | 6 | 2 | 2 | 0.491 | 0.005 | 0.085 | 0.006 | 0.083 | 0.317 |
| **51-75%** | 2 | 9 | 51 | 19 | 5 | 3 |  |  |  |  |  |  |
| **11-50%** | 2 | 5 | 10 | 0 | 0 | 0 |  |  |  |  |  |  |
| **0-10%** | 0 | 0 | 0 | 0 | 0 | 0 |  |  |  |  |  |  |

P1 = primer 1 (ADS 783); P2 = primer 2 (ADS 3576); IFNy+ = enriched IFNy+ Treg preparations, IFNy- = enriched IFNy- Treg preparations.

*Mann-Whitney U test: Treg subsets of patients/healthy controls with ≤75% Foxp3 TSDR methylation compared with those of patients/healthy controls with >75% Foxp3 TSDR methylation. Only p-values <0.01 were considered significant (Bonferroni correction). Because of limited blood sample material for Treg subset isolation, determination of Foxp3 TSDR methylation status was not possible with both primers in IFNy+ and IFNy- Treg preparations of every patient blood sample.
