# Supplementary material for: Helios expression and Foxp3 TSDR methylation of IFNy+ and IFNy- Treg from kidney transplant recipients with good long-term graft function
Source: PLoS One. 2017 Mar 15;12(3):e0173773. doi: 10.1371/journal.pone.0173773 (PMC5351987; doi:10.1371/journal.pone.0173773)
Supplement: S2 Table — (DOCX) [file pone.0173773.s002.docx]

| **Parameter** | **Male patients**  **(n=)** | | **Female patients**  **(n=)** | | **GFR**  **Male/ female**  **P*** | **Crea**  **Male/ female**  **P*** |
| --- | --- | --- | --- | --- | --- | --- |
|  | **Methy-**  **lation**  **≤75%**  **(n)** | **Methy-**  **lation**  **>75%**  **(n)** | **Methy-**  **lation**  **≤75%**  **(n)** | **Methy-**  **lation**  **>75%**  **(n)** |  |  |
| **IFNγ+ Treg Primer P1** | 33 | 30 | 6 | 24 | 0.606/ 0.900 | 0.690/ 0.781 |
| **IFNγ- Treg Primer P1** | 35 | 34 | 7 | 30 | 0.425/ 0.556 | 0.414/ 0.635 |
| **IFNγ+ Treg Primer P2** | 32 | 10 | 14 | 8 | 0.182/ 0.973 | 0.202/ 0.973 |
| **IFNγ- Treg Primer P2** | 60 | 5 | 19 | 6 | 0.479/ 0.106 | 0.784/ 0.106 |

P1 = primer 1 (ADS 783); P2 = primer 2 (ADS 3576); IFNy+ = enriched IFNy+ Treg preparations, IFNy- = enriched IFNy- Treg preparations.

*Mann-Whitney U test: methylation ≤75% vs >75%. Because of limited blood sample material for Treg subset isolation, determination of Foxp3 TSDR methylation status was not possible from every patient blood sample.
