# Supplementary material for: Helios expression and Foxp3 TSDR methylation of IFNy+ and IFNy- Treg from kidney transplant recipients with good long-term graft function
Source: PLoS One. 2017 Mar 15;12(3):e0173773. doi: 10.1371/journal.pone.0173773 (PMC5351987; doi:10.1371/journal.pone.0173773)
Supplement: S3 Table — (DOCX) [file pone.0173773.s003.docx]

| **Infection** | **IFNγ+ Treg**  **Primer P1**  **%Methy-**  **lation** | | **P*** | **IFNγ- Treg**  **Primer P1**  **%Methy-**  **lation** | | **P*** | **IFNγ+ Treg**  **Primer P2**  **%Methy-**  **lation** | | **P*** | **IFNγ- Treg**  **Primer P2**  **%Methy-**  **lation** | | **P*** |
| --- | --- | --- | --- | --- | --- | --- | --- | --- | --- | --- | --- | --- |
|  | **≤75** | **>75** |  | **≤75** | **>75** |  | **≤75** | **>75** |  | **≤75** | **>75** |  |
| **Male patients** | | | | | | | | | | | | |
| **No viral (n)** | 32 | 28 | 0.601 | 32 | 33 | 1.0 | 32 | 10 | 1.0 | 57 | 5 | 1.0 |
| **Viral (n)** | 1 | 2 |  | 2 | 1 |  | 0 | 0 |  | 3 | 0 |  |
| **No bacterial (n)** | 26 | 25 | 0.754 | 26 | 31 | 0.186 | 28 | 7 | 0.328 | 51 | 4 | 0.579 |
| **Bacterial (n)** | 7 | 5 |  | 8 | 3 |  | 4 | 3 |  | 9 | 1 |  |
| **Female patients** | | | | | | | | | | | | |
| **No viral (n)** | 5 | 22 | 1.0 | 7 | 27 | 1.0 | 14 | 8 | 1.0 | 18 | 4 | 0.217 |
| **Viral (n)** | 0 | 0 |  | 0 | 0 |  | 0 | 0 |  | 0 | 1 |  |
| **No bacterial (n)** | 4 | 17 | 1.0 | 6 | 19 | 0.644 | 10 | 2 | 0.758 | 13 | 4 | 1.0 |
| **Bacterial (n)** | 1 | 5 |  | 1 | 8 |  | 3 | 1 |  | 5 | 1 |  |

P1 = primer 1 (ADS 783); P2 = primer 2 (ADS 3576); IFNy+ = enriched IFNy+ Treg preparations, IFNy- = enriched IFNy- Treg preparations.

*Fisher´s exact test: no viral vs viral; no bacterial vs bacterial. Because of limited blood sample material for Treg subset isolation, determination of Foxp3 TSDR methylation status was not possible from every patient blood sample.
