# Supplementary material for: Helios expression and Foxp3 TSDR methylation of IFNy+ and IFNy- Treg from kidney transplant recipients with good long-term graft function
Source: PLoS One. 2017 Mar 15;12(3):e0173773. doi: 10.1371/journal.pone.0173773 (PMC5351987; doi:10.1371/journal.pone.0173773)
Supplement: S4 Table — (DOCX) [file pone.0173773.s004.docx]

| **Rejection** | **IFNγ+ Treg**  **Primer P1**  **%Methy-**  **lation** | | **P*** | **IFNγ- Treg**  **Primer P1**  **%Methy-**  **lation** | | **P*** | **IFNγ+ Treg**  **Primer P2**  **%Methy-**  **lation** | | **P*** | **IFNγ- Treg**  **Primer P2**  **%Methy-**  **lation** | | **P*** |
| --- | --- | --- | --- | --- | --- | --- | --- | --- | --- | --- | --- | --- |
|  | **≤75** | **>75** |  | **≤75** | **>75** |  | **≤75** | **>75** |  | **≤75** | **>75** |  |
| **Male patients** | | | | | | | | | | | | |
| **No rej (n)** | 22 | 21 |  | 24 | 25 |  | 22 | 10 |  | 39 | 2 |  |
| **Rej Banff >1 (n)** | 1 | 0 | 1.0 | 1 | 3 | 0.613 | 4 | 0 | 0.559 | 4 | 0 | 1.0 |
| **Borderline rej (n)** | 4 | 4 | 1.0 | 6 | 2 | 0.258 | 2 | 0 | 1.0 | 7 | 1 | 0.421 |
| **Female patients** | | | | | | | | | | | | |
| **No rej (n)** | 4 | 13 |  | 5 | 17 |  | 13 | 5 |  | 11 | 4 |  |
| **Rej Banff >1 (n)** | 0 | 3 | 1.0 | 1 | 3 | 1.0 | 0 | 0 | 1.0 | 3 | 0 | 0.554 |
| **Borderline rej (n)** | 0 | 3 | 1.0 | 0 | 3 | 1.0 | 0 | 1 | 0.316 | 2 | 0 | 1.0 |

P1 = primer 1 (ADS 783); P2 = primer 2 (ADS 3576); IFNy+ = enriched IFNy+ Treg preparations, IFNy- = enriched IFNy- Treg preparations.

Rej = rejection; *Fisher´s exact test: no rejection vs rejection Banff >1; no rejection vs borderline rejection. Because of limited blood sample material for Treg subset isolation, determination of Foxp3 TSDR methylation status was not possible from every patient blood sample.
